# Closed-loop digital meditation for neurocognitive and behavioral development in adolescents with childhood neglect

**DOI:** 10.1038/s41398-020-0820-z

**Published:** 2020-05-18

**Authors:** Jyoti Mishra, Rajesh Sagar, Sana Parveen, Senthil Kumaran, Kiran Modi, Vojislav Maric, David Ziegler, Adam Gazzaley

**Affiliations:** 1 Department of Psychiatry, University of California San Diego, La Jolla, CA USA; 2 Neural Engineering & Translation Labs, University of California San Diego, La Jolla, CA USA; 3 Department of Psychiatry, All India Institute of Medical Sciences, New Delhi, India; 4 Department of Pediatric Neurology, All India Institute of Medical Sciences, New Delhi, India; 5 Department of Nuclear Magnetic Resonance, All India Institute of Medical Sciences, New Delhi, India; 6 Udayan Care, New Delhi, India; 7 Department of Neurology, University of California San Francisco, San Francisco, CA USA; 8 Neuroscape, University of California San Francisco, San Francisco, CA USA; 9 Weill Institute for Neurosciences & Kavli Institute for Fundamental Neuroscience, University of California San Francisco, San Francisco, CA USA; 10 Department of Psychiatry, University of California San Francisco, San Francisco, CA USA; 11 Department of Physiology, University of California San Francisco, San Francisco, CA USA

**Keywords:** Physiology, ADHD

## Abstract

Adverse childhood experiences are linked to poor attentive behaviors during adolescence, as well as increased risk for mental health disorders in adults. However, no study has yet tested targeted interventions to optimize neurocognitive processes in this population. Here, we investigated closed-loop digital interventions in a double-blind randomized controlled study in adolescents with childhood neglect, and evaluated the outcomes using multimodal assessments of neuroimaging, cognitive, behavioral, and academic evaluations. In the primary neuroimaging results, we demonstrate that a closed-loop digital meditation intervention can strengthen functional connectivity of the dorsal anterior cingulate cortex (dACC) in the cingulo-opercular network, which is critically developing during the adolescent period. Second, this intervention enhanced sustained attention and interference-resolution abilities, and also reduced behavioral hyperactivity at a 1-year follow-up. Superior academic performance was additionally observed in adolescents who underwent the digital meditation intervention. Finally, changes in dACC functional connectivity significantly correlated with improvements in sustained attention, hyperactivity, and academic performance. This first study demonstrates that closed-loop digital meditation practice can facilitate development of important aspects of neurocognition and real-life behaviors in adolescents with early childhood neglect.

## Introduction

Early childhood adversity is associated with a sequelae of cognitive deficits^1–3^. More recent research demonstrates that adversity specifically in the form of neglect, but not abuse, is related to cognitive dysfunction^4–6^. During the adolescent years, such adverse experiences even manifest as attention-deficit hyperactivity disorder (ADHD)^7,8^.

Indeed, adolescence is a critical time for the development of functional brain networks that control attention and related cognition^9,10^. Two important cognitive networks, the cingulo-opercular network and the frontoparietal network, implicated in sustained attentional control vs. moment-to-moment flexible control, are developing during this time^11–14^. Adolescence typically marks the time period for segregation of these networks. In this context, the dorsal anterior cingulate (dACC) has been evidenced as a key cortical region for segregation of networks during adolescence. During this time, the dACC develops stronger functional associations within the cingulo-opercular network, specifically with the anterior insula/frontal operculum region (aI/FO), and weakens its associations to the frontoparietal network^11–16^. The dACC has also been shown to be structurally impacted in children with a history of maltreatment^17^. Yet, the developing functional connections of the dACC in adolescents with childhood neglect are not well-understood. Furthermore, no study has tested targeted interventions that may optimize attention-related neurocognitive processes in this population.

In this collaborative international research, our goal was to evaluate digital closed-loop interventions in adolescents with childhood neglect. Closed-loop interventions delivered in a digital format have two key features—they provide instantaneous feedback on the performance of the individual and they use psychometric functions to continuously adapt to individual performance in order to maximize learning^18,19^. Here, we used multimodal outcomes to evaluate the closed-loop interventions, including functional neuroimaging, complemented with objective assessments of sustained attention and interference resolution, and caregiver-reported inattention/hyperactivity behaviors. Teacher-reported academic performance outcomes were also obtained at post intervention. Study participants were adolescents at a childcare center in India that provides shelter, nurturing, and education access to children who have suffered early life adversity. Notably, the prevalence of childhood maltreatment in India is similar to that observed in the United States^20^, and Indian studies also report similar prevalence for diagnoses of ADHD^21^.

In prior international research in India, we have demonstrated that closed-loop digital interventions that adaptively train attention to goal-relevant information while suppressing sensory distractions, can benefit children with ADHD in this setting^22^. We have further shown that such an intervention has a neural basis in recovering deficient distractor processing^23^. Yet, all such prior work has focused on children living within their biological family homes and with no reported history of early life adversity. Moreover, meta-analyses of game-based digital interventions in children with ADHD do not support a consensus on behavioral outcome benefits^24^. Hence, in order test what may benefit adolescents with neglected childhoods, here, we contrasted two very different digital attention-based interventions, one that targets internal attention to one’s own breath akin to meditation vs. an externally focused approach.

**Fig. 1:**
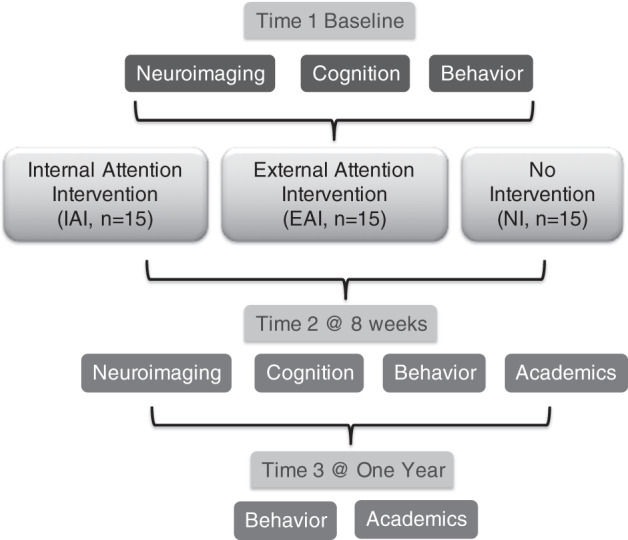
Study design overview. In total, 45 adolescents with a history of childhood trauma completed the study. The study included multidimensional assessments of functional brain networks using neuroimaging, cognitive evaluations, inattention, and hyperactivity behavior ratings provided by caregivers, and teacher-based academic ratings. Assessments were phased at three time points, baseline time 1, post-intervention time 2 (i.e., 8 weeks from baseline), and 1-year follow-up time 3. Adolescents were cluster-randomized into three intervention arms, with double-blind intervention delivery.

Internal attention refers to selective processing of internally generated signals, while external attention refers to the ability to select and modulate incoming sensory information^25^. Extant digital interventions train external attention, as individuals learn to adaptively focus on goal-relevant visual and auditory targets, and suppress irrelevant sensory distractions in game-based environments. In contrast, internal attention is traditionally trained as teacher-guided mindfulness/meditation practices that are nondigital. In one of its basic forms, meditation encourages focus on an internal stimulus anchor such as the breath, while becoming aware and then letting go of interfering thought distractions. Notably, Ziegler et al.^26^ recently implemented this approach in a closed-loop mobile digital meditation program and showed improvements in sustained attention abilities in young adults. Here, we adopted this novel and easily implementable digital strategy to train internal attention in adolescents with childhood neglect. Hence, in this study, we compared an internal attention intervention (IAI) akin to meditation, with an external attention intervention (EAI) that trains attention to sensory visual and auditory stimuli amidst sensory distractions. Our study also included a no-intervention (NI) control. The study followed a double-blind randomized controlled design^27^ (Fig. 1), and both IAI and EAI interventions were delivered in digital closed-loop format on mobile devices in order to match intervention implementation across study arms. Of note, the use of mobile devices for intervention delivery is of great significance for a global mental health setting with limited resources for in-person interventions, as well as for facilitating future scalability^28^.

Many studies that have investigated the neural correlates of mindfulness meditation in adults show plasticity of the dACC^29^, a key brain region for sustained attentional control^30,31^. Given this, here we hypothesized that particularly the IAI digital meditation training will result in functional plasticity of the dACC. Specifically, we hypothesized that dACC functional connectivity to the anterior insula/frontal opercular region, aI/FO, may be modulated, as dACC is expected to connect to aI/FO during adolescent development^11–16^, and this region is also important for both sustained attentional control and self-regulation^9–11,30–34^. Second, we hypothesized that the digital meditation intervention would impart positive changes in objective measures of sustained attention and interference resolution, and improvement in ADHD behaviors. We also hypothesized that intervention group differences would be observed in academic performance.

## Materials and methods

This study has been registered on the International Standard Randomized Controlled Trial Number Registry (ISRCTN94097629)^27^.

### Participants

We recruited 45 adolescents (10–18 years of age, mean age 13.9 ± 1.8 years, 30 males) from a stable childcare center in New Delhi, India to participate in this research; the *Udayan Care* stable care center provides shelter, nurturing, and education access to children who have suffered early life adversity. Ethical approvals for this international study were obtained from the institutional review boards at the University of California San Francisco, the All India Institute for Medical Sciences (AIIMS) New Delhi, the ethical considerations for research committee at *Udayan Care*, as well as the Indian Health Ministry’s Screening Committee, which is the ethical body of the Indian Council of Medical Research that oversees all international collaborative research in India. Participants’ caregivers provided signed informed consent for the research, and all enrolled adolescents provided verbal assent.

### Screening

All study participants completed self-reports on the childhood trauma questionnaire (CTQ^35^) that scores trauma in the domains of emotional and physical neglect and/or emotional, physical, and sexual abuse. We calculated individual CTQ scores as the mean scores across all five abuse/neglect subdomains that are measured on this scale (score across 45 participants, mean 2.08 ± 0.58, range 1.21–3.89). Individual subdomain scores are typically calculated as sum scores across five questions in each subdomain, each question rated on a 1–5 Likert scale; hence, sum scores range from 5 to 25, and mean subdomain sum scores range from 1 to 5. Mean scores in each subdomain were 1.72 (±0.12) for emotional abuse, 1.75 (±0.12) for physical abuse, 1.25 (±0.08) for sexual abuse, 2.70 (±0.13) for emotional neglect, and 2.32 (±0.12) for physical neglect. These scores were “low to moderate” with respect to trauma severity based on validated threshold cutoffs in each subdomain (mean cutoff, emotional abuse ≥ 1.8, physical abuse ≥ 1.6, sexual abuse ≥ 1.2, emotional neglect ≥ 2, and physical neglect ≥ 1.6)^36^. Subdomain mean scores were highly correlated within each domain for abuse (emotional vs. physical *r* = 0.64, *p* < 0.0001, emotional vs. sexual *r* = 0.34, *p* = 0.02, and physical vs. sexual, *r* = 0.36, *p* = 0.02) and for neglect (emotional vs. physical *r* = 0.5, *p* < 0.0001), as well as across abuse and neglect domains (*r* = 0.65, *p* < 0.0001). However, the severity of neglect was significantly greater than severity of abuse (*p* < 0.0001) in our sample. Henceforth, we use childhood neglect as the specific subdomain variable of interest. Participants did not have any comorbid psychiatric conditions or drug abuse history as assessed in clinical interviews.

### Assessments

We conducted multidimensional assessments in all study participants, including neuroimaging as the primary assessment, and complementary secondary assessments, i.e., computerized objective cognitive assessments, caregiver-based ADHD behavioral ratings, and teacher-based academic performance ratings. Neuroimaging and cognitive assessments were performed at two time points 8 weeks apart (time 1 and time 2), interspersed by the intervention period. Behavior ratings were performed at time 1, time 2, and additionally at a 1-year follow-up, time 3. Academic performance ratings could not be obtained at time 1, but were obtained at time 2 and time 3. The overall study design is depicted in Fig. 1.

#### Neuroimaging

Resting-state functional magnetic resonance imaging (rs-fMRI) provides a measure of spontaneous, intrinsic brain activity that can be used to assess multiple functional brain networks^37,38^. We implemented rs-fMRI in this study as it is more feasible in this pediatric population, and also produces larger, more robust, and reliable brain signals of energy consumption than task-based approaches^39–42^. Scans were conducted at AIIMS New Delhi, on a 3.0 T MR Scanner (Phillips Ingenia) equipped with a 32-ch head coil. Scans were performed in 44 of the 45 study participants; one participant was excluded due to dental braces that produce large artifacts during scanning. Anatomical T1-weighted images were collected using a high-resolution 3D magnetization-prepared rapid gradient echo sequence with 360 1-mm-thick sagittal slices (echo time [TE] 3.7 ms; repetition time [TR] 8.1 ms; field of view [FOV] 240 mm; flip angle 8°). Rs-fMRI images were acquired at rest with eyes open, gaze at a central fixation cross, using single-shot echo-planar T2*-weighted imaging sequence. Each volume consisted of 35 contiguous 4-mm-thick slices with ascending slice order and no interslice gap (TE 30 ms; TR 2000 ms; FOV 230 mm; flip angle 90°; duration 6.83 min). The scan duration we used was optimized for obtaining a sufficient number of reliable scans in pediatric populations^43–45^, which consisted of 200 volumes plus 5 initial unscored dummy volumes acquired in 6.83 min.

Rs-fMRI data were preprocessed in SPM12 (The Wellcome Department of Cognitive Neurology, London, UK, http://www.fil.ion.ucl.ac.uk/spm/software/spm12/) using standard spatial preprocessing steps. Functional data were slice-time corrected, realigned to the first image of the resting scan, normalized in Montreal Neurological Institute (MNI) space, and smoothed with a 6-mm kernel (full width at half maximum). Functional connectivity analysis was performed using a seed-driven approach using the CONN toolbox v17 (http://www.nitrc.org/projects/conn)^46^.

Physiological and other spurious sources of noise were estimated and regressed out using the anatomical CompCor method (aCompCor) that has been shown to yield higher specificity and sensitivity compared with global signal regression^47,48^. A temporal band-pass filter of 0.008–0.09 Hz was applied simultaneously to all regressors in the model. Residual head motion parameters (three rotation and three translation parameters plus another six parameters representing their first-order temporal derivatives) were regressed out. Artifact/outlier scans (average intensity deviating more than three standard deviations from the mean intensity in the session or composite head movement exceeding 1 mm from the previous image) were also regressed out to minimize the spurious effects induced by motion artifacts^49^. Outlier images were modeled as nuisance covariates. Each outlier image was represented by a single regressor in the general linear model (GLM), with a 1 for the outlier timepoint and 0 elsewhere. We confirmed that head displacement for either frame-to-frame translations or rotations did not significantly differ across time 1 and 2 scans (mean ± standard error of *xyz* translations, time 1: 0.05 ± 0.007 mm, time 2: 0.05 ± 0.004 mm; rotations, time 1: 7 × 10^−4^ ± 2 × 10^−4^ radians, time 2: 6 × 10^−4^ ± 8 × 10^−5^ radians). The number of outliers also did not significantly differ between time 1 and 2 scans (time 1: 19 ± 3, time 2: 18 ± 3). We additionally confirmed that when participants are partitioned into intervention groups, that there are no significant differences in head displacement parameters or outlier scans between intervention groups (*p* > 0.5), nor any intervention group × time interaction (*p* > 0.25).

We analyzed the dACC seed region connectivity to both cingulo-opercular and frontoparietal network regions of interest (ROIs) as dACC is found to be functionally connected to several of these regions during development^11–16^. The dACC seed region was defined as a 12-mm radius sphere around peak coordinates (MNI *x*, *y*, *z*: −2, 7, 50); 11 ROIs were similarly specified in the frontoparietal network (right/left precuneus, mid cingulate, right/left dorsolateral prefrontal cortex, right/left frontal cortex, right/left inferior parietal lobule, and right/left intraparietal sulcus) and 6 ROIs specified in the cingulo-opercular network (right/left anterior insula/frontal operculum (aI/FO), right/left anterior thalamus, and right/left anterior prefrontal cortex) as per previously described ROI coordinates^11,31^. Time series of all voxels within each ROI were averaged, and first-level correlation maps were produced by extracting the residual BOLD signal time course from the dACC seed ROI and computing Pearson correlation coefficients between its time course and the time course of all other ROIs. Correlation coefficients were converted to normally distributed *Z* scores using the Fisher transformation to allow for second-level GLM analyses. Mean functional connectivity strengths of the dACC to the frontoparietal network and to the cingulo-opercular network were calculated as the mean functional connectivity between the dACC seed and the 11 frontoparietal network ROIs and the 6 cingulo-opercular network ROIs, respectively.

In addition, we performed seed–voxel correlations by estimating maps showing temporal correlations between the BOLD signal from the dACC seed region and the time course of all other voxels. Pearson correlation coefficients were converted to normally distributed *Z* scores using the Fisher transformation to allow for second-level GLM analyses. For this seed–voxel connectivity data, cluster-level threshold was set at *p* < 0.05 using false-discovery rate correction for multiple comparisons, with voxelwise threshold of *p* < 0.01^50^.

#### Objective cognitive assessments

We tested participants on two standard objective cognitive assessments that are frequently tested in children with ADHD, evaluating (a) sustained attention to goal-relevant information, modeled after the test of variables of attention^51^, and (b) interference resolution as per the Flanker test^52^. In the sustained attention test, participants detected sparse targets (black square in the visual upper field appearing on 33% of trials) and withheld responses on frequent nontargets (black square in the visual lower field appearing on 67% of trials). Accuracies on this task are typically at ceiling, and the relevant response measure is the response time variability, with lower response variability indicative of higher consistency and better performance^53^. In the Flanker test for interference resolution, participants viewed an array of five letters and identified the central target letter while ignoring the flanking distractor letters. The letters, a/b/c/d were used, with each serving as a target or flanking letter on an equivalent number of trials. In all, 50% of task trials were congruent with matching targets and distractors, and 50% were incongruent with different targets and distractors. The main performance measure on this task is the response time cost (i.e., RT on incongruent trials minus RT on congruent trials), with smaller response time costs indicative of better performance.

#### Behavior ratings

Caregivers rated inattention and hyperactivity behaviors on the standard ADHD-RS IV rating scale^54^ that has also been previously used in research in India^21,22^. The same caregiver for each child rated ADHD behaviors at time 1–3. Raw scores, clinically normed percentiles, as well as the number of adolescents that surpassed clinical 80% threshold are summarized in Table 1.

**Table 1:** Participant characteristics and outcome measures.

Measure	IAI	EAI	NI	Group difference
Gender	7F/8M	6F/9M	2F/13M	*p* = 0.13
Age in years	13.8 ± 0.5	13.3 ± 0.4	14.5 ± 0.5	*p* = 0.18
Age of stable care access in years	9.7 ± 0.8	7.8 ± 0.7	6.5 ± 0.4	*p* = 0.006*
Childhood trauma score	2.23 ± 0.11	2.22 ± 0.18	1.80 ± 0.13	*p* = 0.06
Childhood trauma abuse subscore	1.74 ± 0.16	1.63 ± 0.16	1.35 ± 0.12	*p* = 0.18
Childhood trauma neglect subscore	2.65 ± 0.11	2.69 ± 0.26	2.19 ± 0.17	*p* = 0.13
*rs-fMRI dACC-aI/FO connectivity*				
T1	0.40 ± 0.05	0.56 ± 0.06	0.52 ± 0.05	*p* = 0.12
T2	0.51 ± 0.04	0.52 ± 0.05	0.51 ± 0.06	*p* = 0.03*
*Sustained attention*				
T1	150 ± 17	129 ± 10	141 ± 10	*p* = 0.70
T2	107 ± 8	203 ± 36	222 ± 35	*p* = 0.01*
*Interference resolution*				
T1	97 ± 24	55 ± 16	33 ± 8	*p* = 0.07
T2	29 ± 13	47 ± 11	27 ± 10	*p* = 0.03*
*Inattention ratings*				
T1	7.7 ± 1.8 (75 ± 19% 6 of 15)	9.2 ± 1.6 (80 ± 19% 8 of 15)	7.3 ± 1.4 (75 ± 19% 6 of 15)	*p* = 0.68
T2	6.2 ± 1.3(75 ± 18% 4 of 15)	7.9 ± 1.2 (80 ± 11% 8 of 15)	10.4 ± 1.3 (84 ± 9% 9 of 15)	*p* = 0.07
T3	6.2 ± 1.5(75 ± 19% 4 of 13)	9.4 ± 1.7 (86 ± 8% 8 of 13)	7.5 ± 1.3 (80 ± 7% 6 of 12)	*p* = 0.40
*Hyperactivity ratings*				
T1	7.5 ± 1.3 (84 ± 10% 10 of 15)	7.1 ± 1.0 (75 ± 9% 7 of 15)	8.4 ± 1.2 (84 ± 9% 10 of 15)	*p* = 0.77
T2	4.7 ± 1.0 (75 ± 12% 5 of 15)	5.7 ± 1.0 (75 ± 17% 6 of 15)	7.5 ± 1.0 (87 ± 7% 10 of 15)	*p* = 0.12
T3	2.3 ± 0.6 (50 ± 25% 0 of 13)	6.2 ± 1.5 (75 ± 20% 6 of 13)	5.8 ± 1.2 (75 ± 17% 5 of 12)	*p* = 0.01*
*Academic performance*				
T2	65 ± 4	58 ± 2	58 ± 2	*p* = 0.04*
T3	57 ± 4	53 ± 2	57 ± 3	p = 0.64

Measures of participants in the IAI (internal attention intervention), EAI (external attention intervention), and NI (no intervention) study arms at baseline alone, or as measured at baseline (T1), post intervention (T2), and 1-year follow-up (T3). Data measure with continuous values are reported as mean ± standard error. Sustained attention was measured by the standard deviation of responses in milliseconds on a continuous performance task; interference resolution was measured as the response time cost difference in milliseconds for responding to stimuli with conflict vs. no conflict on a Flanker task. For inattention and hyperactivity ratings, raw measures are accompanied by data in parentheses that are clinically normed percentiles reported as median ± median absolute deviation, followed by the number of adolescents that surpassed clinical 80% threshold of the group total. For academic performance, raw scores were out of a max score of 95. For normally distributed measures, group differences were compared using one-way ANOVA at baseline or repeated measures ANOVA at T2 vs. T1 with baseline covariates, else the nonparametric Kruskal–Wallis test was used (see “Materials and Methods”, “Data analyses”). * denotes significant group differences. Significant baseline group differences were only observed for age of stable care access (post hoc, IAI > NI *p* = 0.005, and no other differences). *Abbreviations*: *F* female, *M* male, *rs-fMRI* resting-state functional magnetic resonance imaging, *dACC* dorsal anterior cingulate cortex, *aI/FO* anterior insula/frontal operculum.

#### Academic performance

Teachers rated academic performance on the academic performance rating scale (APRS^55^) at time 2, and different teachers rated performance at time 3; these ratings could not be obtained at time 1. On the APRS, the teacher rates the child’s math, reading, writing, and oral abilities, both in terms of accuracy and consistency. These ratings had some missing data (8 of 45 participants’ missing data at time 2, and 10 of 45 participants’ missing data at time 3).

### Intervention

After baseline assessment time 1, participants were cluster-randomized into either the IAI, EAI, or no Intervention (NI) arms. Cluster randomization was based on enrollment in pre-existing after-school groups. The 45 study participants were pre-enrolled in 6 separate after-school groups based on common gender and age, and common home area. Hence, we randomized two after-school groups each to the IAI (*n* = 15, 7 female, 8 male), EAI (*n* = 15, 6 female, 9 male), and NI (*n* = 15, 2 female, 13 male) intervention arms.

This sample size of 15 participants per study arm (IAI/EAI/NI) for a total sample of 45 participants was sufficiently powered to obtain a large effect size (*η*^2^ ≥ 0.14^56^) between-group (IAI/EAI/NI) effect on the primary outcome, i.e., change in functional connectivity of the dACC to the frontal opercular aI/FO region in the cingulo-opercular network, using a repeated measures analysis of variance (rm-ANOVA) with two repeated measures (baseline vs. post intervention), powered at 0.8 with alpha level of 0.05. A sample size by power plot for this calculation obtained using the G*Power tool^57^ is shown in supplementary figure S1.

Both IAI and EAI were self-administered as digital tablet apps for up to 30 min of practice per session (25 min of training interspersed with short 1-min breaks every 5 min), for 30 sessions over 30 nonconsecutive days (~6 weeks). Participants had no prior exposure to IAI/EAI, or interventions of this kind. To facilitate full adherence and troubleshoot any technical issues, a research staff member was present during all after-school group-training sessions; participants sat in a group, yet, performed their individual training sessions. As a result, all participants in the IAI and EAI arms had 100% intervention adherence. Participants in the NI arm were not provided any intervention, and went about their daily activities as usual between time 1 and time 2 assessments.

The intervention arms were double-blinded; both IAI and EAI arms were experimental; hence, neither the participants nor the research staff interacting with the participants had any knowledge as per the relative efficacy of one or the other arm. Also, the NI arm did not have knowledge of the other IAI/EAI arms, thereby, equating placebo effects in all study arms as much as possible. Caregivers had knowledge that their child was enrolled in a digital intervention, but were blind to the goals of the intervention. Teachers were also intervention-blind, i.e., without any knowledge if a child was participating in this intervention study. At the end of each intervention session, progress and performance data were automatically transferred to a secure study data server in de-identified format.

Participants in the IAI group practiced attending to the sensations of their breath, with monitoring guided using a digital app—*Meditrain*—which recently showed benefits on sustained attention in healthy young adults^26^. Participants were instructed to acknowledge internally distracting thoughts when they occurred during the practice, then disengage from the thought and shift their attention back to their breath. Participants practiced attention to breath in a closed loop, i.e., performance-adaptive trial durations starting as short as 10 s and progressively built up to several minutes of breath focus. Trial durations were adapted based on end-of-trial feedback from participants. At the end of each trial, participants were prompted to report, via a screen-tap, whether their attention remained on their breath throughout the trial, or if their attention was diverted by distracting thoughts. If they reported successful attention to their breath for the entire trial, the duration (in seconds) of the next trial was increased by 10%; if unsuccessful, the duration of the next trial was decreased by 20%. Using this adaptive algorithm, the intervention targeted the participants’ ability to self-regulate internal attention on an individualized basis. Intervention sessions were linked, such that the next session began at the level (i.e., trial duration) attained at the end of the previous session. The IAI participants predominantly reported successful attention to breath (reported percent success mean ± standard error 92.9 ± 0.96%; range 85.3–98.3%) with no significant differences in these percent reports in the first half (sessions 1–15) vs. second half (sessions 16–30) of the intervention (*p* > 0.8). Given the closed-loop nature of the program that lengthens the duration of the breath awareness period after each success, trial durations were significantly longer at the end relative to mid-intervention (mid-intervention trial duration: 93 ± 13 s, end-of-intervention: 793 ± 64 s; *p* < 0.0001).

Participants in the EAI group practiced attention to sensory (visual and auditory) stimuli amidst sensory distractors in the context of five different game modules, practiced 5 min each per session. All EAI modules were closed loop, i.e., performance-adaptive and adjusted task difficulty so that participants maintained ~80% performance accuracy at all times. Game modules challenged focused attention, divided selective attention, as well as working memory when a given sensory target had to be retained amidst varied distractors over several trials. The *Freeze Frame*, *Double Decision, Mind’s Eye*, and *Target Tracker* visual game modules, and the *Hear Hear* and *Memory Grid* auditory modules available at brainhq.com were selected for the EAI based on demonstrated efficacy of these individual training modules in prior research^22,23,58–60^. In *Freeze Frame*, participants practiced focused attention to visual targets, selectively withholding their response to these while non-selectively responding to all distractors. In *Double Decision*, participants practiced divided attention to central and peripheral visual targets. In *Mind’s Eye*, participants identified visual targets amidst simultaneous visual distractors as the features of the visual distractors adaptively resembled the visual target over successive trials. In *Target Tracker*, participants attended to moving object targets amidst moving distractors, and were adaptively challenged to retain a larger number of target objects in working memory. In *Hear Hear*, participants attended to target sounds within sequences of distractor sounds that adaptively resembled the target sound over successive trials. In *Memory Grid*, participants matched pairs of sound clips shuffled among a set of several sound clips of adaptively increasing set size.

### Data analyses

Intervention effects in the primary neuroimaging and secondary cognitive assessments were analyzed in SPSS software using general linear modeling, specifically, group × time repeated measures ANOVAs with between-group factor of intervention (IAI, EAI, and NI) and within-group factor of assessment time (time 1 and 2). All rm-ANOVAs, including baseline covariates of childhood, neglect severity, age, gender, and age at which stable childcare access was obtained to control for differences in these variables across participants; rm-ANOVA group × time interactions were also verified that they did not differ in the absence of covariates. Estimates of effect size were reported as eta squared calculated as the ratio of the sum of squares for the effect relative to the corrected total sum of squares in SPSS (*η*^2^ < 0.06 is small, 0.06–0.14 are medium, and ≥0.14 are large effect sizes^56^). Post hoc testing used two-tailed paired *t* tests.

Intervention effects on ADHD behavioral ratings were analyzed using the nonparametric Kruskal–Wallis test, systematically investigating between-group (IAI, EAI, and NI) differences at time 1, time 2, and at the 1-year follow-up, time 3. Post hoc testing used two-sided Wilcoxon signed rank tests. The Kruskal–Wallis test was also used to investigate between-group (IAI, EAI, and NI) differences in teacher-based academic performance rating raw scores at time 2 and time 3.

For equivalent representation of results across the multidomain assessments (i.e., functional connectivity in rs-fMRI, cognitive performance, and behavioral and academic ratings), individual data were converted to *Z* scores within each domain. *Z* scores were calculated relative to the mean and standard deviation of the baseline (time 1) assessment data across all participants (*n* = 45) in each of the neuroimaging, cognitive, and behavioral domains. For academic data, *Z* scores were independently calculated at time 2 and 3, since baseline time 1 data were absent, and different teachers provided the ratings at time 2/3.

## Results

### No baseline differences between intervention arms

The IAI, EAI, and NI study arms did not differ in any of the neurocognitive and behavioral characteristics measured at baseline (Table 1), except in age at which participants accessed stable care (i.e., a secure environment in which no further childhood trauma occurred). Neither mean CTQ scores, nor abuse/neglect subdomain scores, differed significantly between the IAI, EAI, and NI groups (Table 1). Across all participants, childhood trauma severity significantly correlated with age of stable care access (*r* = 0.31, *p* = 0.04, but not actual age, *p* = 0.09), and girls reported greater childhood trauma than boys (*F*(1,43) = 5.54, *p* = 0.02). To control for variability in baseline age, gender, age of stable care access, and neglect severity, all intervention outcome analyses carried out below accounted for these variables as model covariates.

### Intervention-related plasticity of dACC functional connectivity

**Fig. 2:**
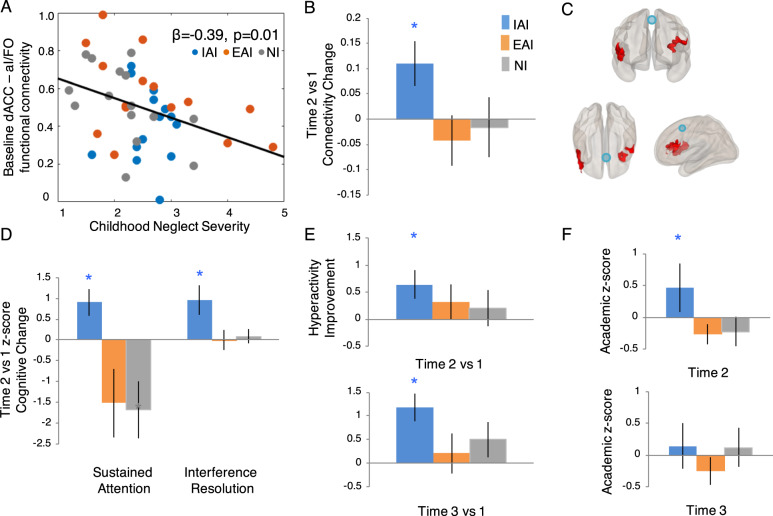
Study outcomes. **a** At baseline, childhood neglect severity was negatively associated with mean dACC connectivity to the anterior insula/frontal operculum (aI/FO) regions in the developing cingulo-opercular network across all participants. **b** dACC connectivity to aI/FO significantly enhanced at time 2, only for the IAI group. **c** Seed–voxel group × time analyses (dACC seed in blue, aI/FO voxels in red) confirmed the result for the ROI–ROI analyses, showing enhanced connectivity in IAI vs. EAI/NI. **d** Cognitive changes at time 2 vs. time 1 showed improvements for IAI vs. EAI/NI for both sustained attention (i.e., reduced response time variance at post intervention, plotted as the time 1 minus 2 difference) and interference resolution (i.e., reduced interference response cost at post intervention, also plotted as the time 1 minus 2 difference). **e** Hyperactivity ratings continued to improve for IAI vs. EAI/NI at the 1-year follow-up, time 3 (plotted as the *Z*-score difference for baseline time 1 minus time 2 (*top*) or time 1 minus time 3 ratings (*bottom*)). **f** Teacher ratings of academic performance were significantly higher for IAI vs. EAI/NI at time 2.

At baseline, childhood neglect severity was significantly negatively associated with dACC mean functional connectivity in the cingulo-opercular network (partial regressions controlling for factors of age, gender, and age of stable care access, *ß* = −0.42, *p* = 0.006, CI: [−0.64 to 0.14]). Within this network, dACC specifically makes strong functional connections with the anterior insula/frontal opercular aI/FO region during adolescent development^11–16^; we found that childhood neglect was negatively associated with this dACC-aI/FO bilateral mean functional connectivity (*ß* = −0.39, *p* = 0.01, CI: [−0.59 to 0.17], Fig. 2a). The association for childhood neglect vs. dACC mean connectivity to the frontoparietal network was not significant (*ß* = −0.18, *p* = 0.26, CI: [−0.45 to 0.13]); this was as expected since dACC separates from the frontoparietal network during adolescence^11–16^. dACC functional connectivity in these networks also did not show any significant correlations with childhood abuse.

We analyzed baseline vs. post-intervention (i.e., time 1 vs. time 2) effects on bilateral dACC-aI/FO connectivity, and found a significant group × time interaction (*F*(2,37) = 4.05, *p* = 0.03, *η*^2^ = 0.14). Post hoc tests revealed that this interaction was driven by significantly enhanced connectivity at time 2 vs. time 1 in the IAI group (*t*(13) = 2.52, *p* = 0.025, CI: [0.02–0.20]), but no significant connectivity change in the EAI and NI groups (*p* > 0.05) (Fig. 2b). These results also replicated in dACC seed-to-voxel whole-brain group × time statistics in which the bilateral aI/FO clusters emerged as the only significant clusters (Fig. 2c).

### Intervention-related changes in cognition

We found significant group × time intervention effects for sustained attention (*F*(2,38) = 5.05, *p* = 0.01, *η*^2^ = 0.17), as well as for interference resolution (*F*(2,37) = 3.70, *p* = 0.03, *η*^2^ = 0.16). Post hoc analyses showed that these cognitive outcomes paralleled the neuroimaging results; only the IAI group significantly improved at time 2 vs. time 1 (sustained attention: *t*(14) = 2.81, *p* = 0.01, CI: [0.21–1.59]; interference resolution: *t*(14) = 2.75, *p* = 0.015, CI: [0.21–1.71]). Changes in the EAI group did not reach significance. The NI group also did not show a significant change for interference resolution, but did show a significant decline in sustained attention at post intervention (*t*(14) = 2.45, *p* = 0.03, CI: [−0.21 to 3.13], Fig. 2d); this poor performance in NI for sustained attention was perhaps due to lack of interest/focus in a repeat assessment that is simple and boring by its very nature^51^.

### Intervention-related changes in inattention and hyperactivity

Caregivers rated inattention and hyperactivity at time 1, time 2, and at the 1-year follow-up time 3. Inattention ratings had no significant group differences at any timepoint. Notably, group differences in hyperactivity ratings emerged at the 1-year follow-up (*H*(2,38) = 9.24, *p* = 0.01, *η*^2^ = 0.23). Post hoc testing showed significant hyperactivity improvements at time 3 relative to baseline, exclusively in the IAI group (signed rank test, *Z* = 75, *p* = 0.005). Consistent with this, hyperactivity ratings also had significantly improved at time 2 vs. time 1 only in the IAI group (*Z* = 19.5, *p* = 0.038), although between-group significance was not yet achieved at time 2.

### Intervention-related academic performance

Academic performance ratings were first feasibly obtainable from teachers at post-intervention time 2; these showed significant group differences, with higher ratings for the IAI relative to EAI and NI groups (*H*(2,37) = 6.26, *p* = 0.04, *η*^2^ = 0.15). These group differences, however, did not reach significance at the 1-year follow-up time 3, when different teachers rated academic performance (Fig. 2f).

### Associations between neural and cognitive/behavioral outcomes

**Fig. 3:**
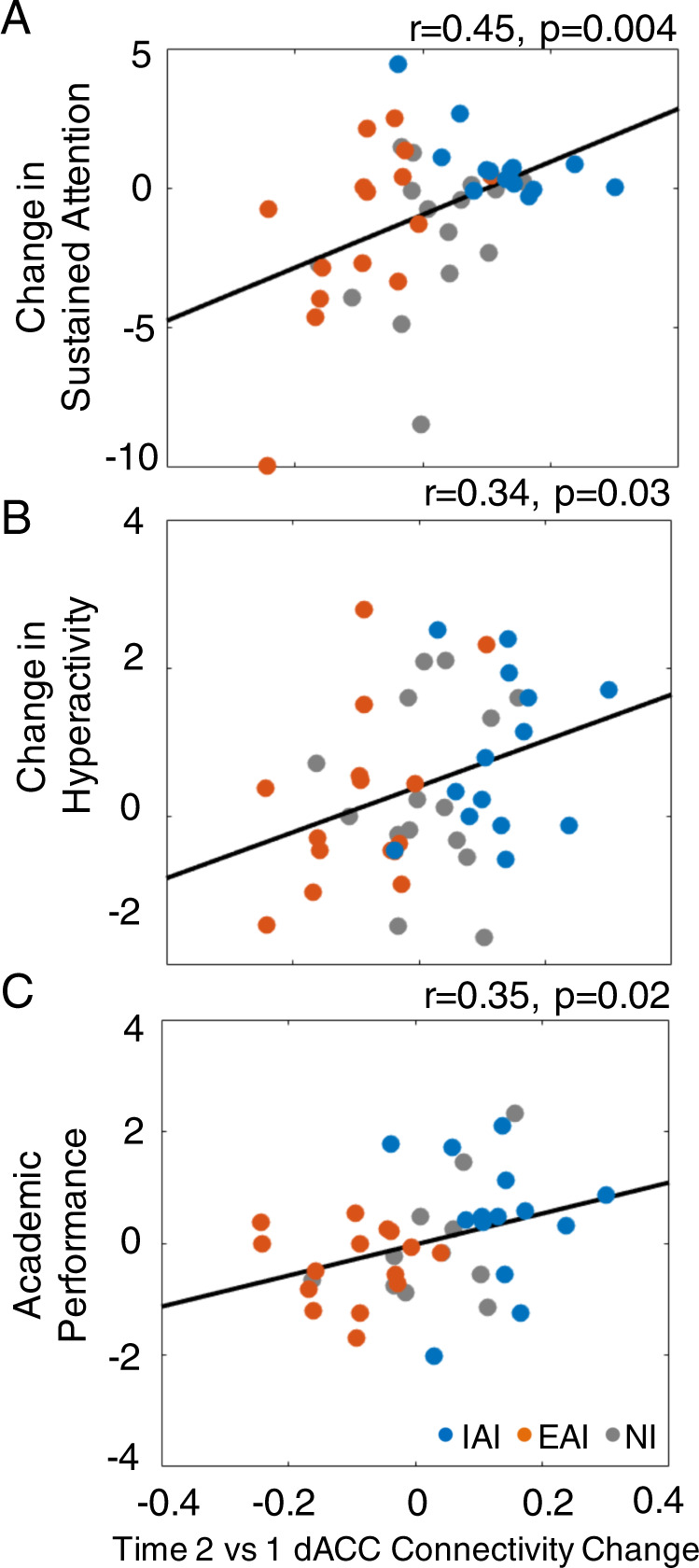
Neurobehavioral correlates. Correlations between change (at time 2 vs. 1) in dACC connectivity to bilateral anterior insula/frontal operculum regions and outcomes across all participants, specifically **a** change in sustained attention performance, **b** change in hyperactivity, and **c** academic performance. Outcomes are depicted in *Z* scores with positive values indicating better outcomes.

Finally, to investigate whether neural and cognitive/behavioral changes are related, we performed Pearson’s correlations between functional connectivity and cognitive/behavioral outcomes across all participants (i.e., including all three groups). These showed that time 2 vs. time 1 changes in dACC-aI/FO functional connectivity were positively correlated with changes in sustained attention (*r* = 0.45, *p* = 0.004, CI: [0.13–0.67]). The relationship between functional connectivity changes and interference-resolution outcomes was not significant. Changes in functional connectivity also significantly related to the median change in hyperactivity outcomes at time 2/3 (*r* = 0.34, *p* = 0.03, CI: [0.07–0.60]) as well as median academic outcomes at time 2/3 (*r* = 0.35, *p* = 0.02, CI: [0.10–0.56]) (Fig. 3).

## Discussion

In this study, we aimed to understand how adolescents affected by early childhood adversity, particularly neglect, may benefit from scalable digital interventions to help facilitate neurocognitive development. The research is novel in being a double-blind randomized controlled study of digital interventions using multidimensional outcomes—neuroimaging, cognition, child behaviors, and academic performance, and instantiated as a global mental health collaboration. Here, we find evidence that severity of early childhood neglect is negatively associated with the strength of functional connections from the dACC, an important cognitive control region. We, further, show that a targeted digital intervention that simulates basic meditative practice, can enhance these functional connections, along with positively impacting broader outcomes in the domains of cognition and hyperactive behaviors; higher academic performance ratings are also observed in the digital meditation group.

Adolescence is a critical time period for both structural and functional brain development, especially for frontal and parietal brain networks that underlie cognition^9–16,32^. Brain networks are understood to be segregating into their selective roles during this time period, together with active cross-network communication. This network segregation is likely very important as adolescents undergo their stressful transition to fully independent adults^61^. Analyses of several task-based fMRI studies show that the cingulo-opercular and frontoparietal networks are distinct cognitive control networks in adults, responsible for stable, sustained control vs. moment-to-moment flexible control, respectively^31,32,34^. Resting-state fMRI studies corroborate these results, and further demonstrate that resting-state functional connectivity is relatively better at predicting functional network maturity than task-based imaging^13^.

While functional network development in typical adolescence is increasingly understood, little is known about the development of cognitive control networks in adolescents with a history of childhood adversity. From a huge literature on adult retrospective studies, we understand that these individuals demonstrate scholastic underachievement, school dropout, and juvenile delinquency, are vulnerable to mental illness, and have alcohol and substance abuse problems that are all precursors to a lifetime of instability^62,63^. Notably, in a recent large-sample adolescent study conducted on a National Institute of Health dataset, Silveira et al.^64^ demonstrate that functional connectivity in distributed developing brain networks is impacted by childhood adversity; these functional networks include the important dACC region, and in turn mediate poor executive function and predict alcohol abuse in future years. This research dovetails with our neural findings in this international cohort, which also shows that dACC functional connectivity is negatively associated with the severity of childhood neglect. Of note, these are now convergent findings across developing adolescent brain networks in the United States and in India.

The dACC is a crucial cognitive control network region that has a core role in sustained attention processing, as well as in resolution of interference^33,65^. Frontal theta oscillations measured in human electrophysiology, localize to this region, and have been suggested as the biophysical basis of brain network communications emergent from the dACC^66^. That dACC functional connectivity is impacted in adolescent brains that have experienced early adversity, is concerning for the development of the fundamental abilities of selective attention and interference resolution.

Here, for the first time, we systematically test scalable, attention-targeted digital interventions in adolescents with childhood neglect. In a three-arm, double-blind, cluster-randomized study, we test internal vs. EAIs vs. a NI (life-as-usual) control. Of note, both active intervention arms demonstrated 100% adherence to their training programs, attesting to the feasibility of engaging digital interventions in these adolescents. The training focus of the IAI was akin to meditation, anchored to the individual’s own breath, while encouraging suppression of internal distractions. Uniquely, the closed loop, i.e., performance-adaptive nature of this meditative program is an important research advance that facilitated feasibility, with initial practice times as short as 10 s at a time.

Recently, Ziegler et al.^26^ demonstrated that the digital meditation intervention induces benefits in sustained attention abilities in healthy young adults; specifically, it reduced response variance on the sustained attention test in young adults, a finding that we replicate here in adolescents with neglect. Our studies also find complementary results in the context of interference resolution; here, we measure the relative interference cost for flanking congruent vs. incongruent distractors, and show enhanced performance after digital meditation, while Ziegler et al. showed that visual object discrimination amidst simultaneous object distractions was improved by this treatment. Notably, we did not observe these results in the EAI cohort that focused on computerized visual and auditory trainings, or in the NI cohort. These findings suggest the targeted utility of training internal attention in this vulnerable population.

Beyond immediate cognitive outcomes, we also show that the IAI is associated with significantly enhanced functional connectivity of the dACC-aI/FO brain regions. This finding is in line with studies evidencing functional gains in dACC after traditional meditative training practices^29^. Our finding also parallels the results obtained by Ziegler et al.^26^ who showed that EEG-based theta band (4–8 Hz) coherence, which localizes to mid–frontal regions that include the dACC, is enhanced by digital meditation in young adults.

With respect to developing behaviors, we found that caregivers, blind to the interventions, rated significantly reduced hyperactivity only in the IAI group. This effect showed between-group significance at the 1-year follow-up, and was also observed at the within-group level post intervention; of note, the number of adolescents in the IAI group that surpassed clinical thresholds for hyperactivity fell to zero at the 1-year follow-up. Improvement in hyperactivity is particularly important, given that meta-analyses of several prior digital cognitive trainings in ADHD have concluded null effects on hyperactivity^24^. In addition, behavioral gains at the follow-up timepoint are consistent with our prior results on training programs in children with ADHD, which showed that such gains usually appear subsequent to plasticity in neurocognitive outcomes^22^. Sustainable gains at a 1-year follow-up also compellingly suggest that the IAI group may have learned a generalized self-regulation strategy of internal focus and distractor suppression that is attributed to emerge from dACC function^30,33^. These individuals may continue to practice internal attention even after the formal digital intervention period (i.e., without any digital device access), a possibility that remains to be formally confirmed in future research.

Finally, we observed that teacher-rated academic performance at post intervention was differentially greater in the IAI cohort. This is a limited observation as academic outcomes could not be obtained at baseline, and ratings obtained from different teachers at the 1-year follow-up did not achieve group-level significance. Nevertheless, the significant correlative association of these academic ratings with changes in dACC functional network connectivity, suggests important linkages between these outcomes, which should be systematically pursued in future work. Plasticity of the dACC functional network also correlated with the cognitive outcomes of sustained attention and behavioral outcomes of reduced hyperactivity, further suggesting an underlying mechanism for the cognitive and behavioral findings.

Overall, this study shows that developing attentive self-focus with the breath as an anchor, in theory a simple practice inspired by ancient practices of focused meditation, can have wide-ranging positive effects on important functional brain networks, cognition, and real-life behaviors in adolescents with a history of adversity. Notably, our results show large effect sizes on the primary outcome of dACC-aI/FO functional connectivity, as well as on the secondary cognitive and behavioral outcomes. There are only a handful of studies in the literature on meditation practices in children/adolescents and even fewer with rigorous double-blind methodology^67^; none of them have implemented multimodal evaluations, or used closed-loop digital formats, or investigated outcomes in at-risk adolescents with a history of neglect, as in this study. Furthermore, meditation and mindfulness practices can have varied forms, from focusing on the breath as an anchor to other focused meditation strategies, complex sequences of nostril breathing as in *pranayama*, body scans, walking, meditation, and full-body yoga practices. Here, we employ a basic form of breath-focused meditation throughout, which allows us to gain mechanistic insights that cannot be gleaned by combining multiple meditative approaches. Notably, the dACC locus of control that we demonstrate is consistent with the prior literature on meditation, as well as neurophysiological observations showing specific functional and anatomical links between the brainstem breathing control centers and the ACC/insula^68^.

The limitations of this first study include the need for replication in future large-sample research. Our sample was also heterogeneous in terms of meeting clinical diagnostic criteria for ADHD; hence, the study cannot establish direct clinical relevance to this disorder. Given our specific hypothesis focused on attention-related brain networks, and emergent cognition and behaviors, we also did not track symptoms of other clinical diagnoses, such as depression/anxiety. In terms of data for the digital interventions, it was useful in confirming that all participants completed all training sessions (100% adherence was observed), yet, in exploratory analyses, session-to-session performance metrics did not significantly relate to any intervention outcome measures; hence, future digital intervention versions need to revise these session performance metrics to glean better insights on treatment mechanisms. Finally, our findings are limited to the specific digital interventions we have studied, and cannot generalize to other forms of IAIs/EAIs.

In conclusion, here we show novel utility of a closed-loop digital intervention that trains internal attention^26^, and demonstrates its efficacy in an initial randomized controlled study. The simplicity of the intervention approach is such that it can become integral to daily-life practice and potentially sustain mental health well beyond the initial training period. Importantly, the innovative digital implementation allows for feasible and cost-effective scaling to limited-resource settings, which can help promote well-being of children and youth in our modern digital world.

## Supplementary information

Supplementary Information
