# Supplementary material for: Closed-loop digital meditation for neurocognitive and behavioral development in adolescents with childhood neglect
Source: Transl Psychiatry. 2020 May 18;10:153. doi: 10.1038/s41398-020-0820-z (PMC7235252; doi:10.1038/s41398-020-0820-z)
Supplement: Supplementary file 1 — Supplementary Information [file 41398_2020_820_MOESM1_ESM.pdf]

# Closed-loop digital meditation for neuro-cognitive and behavioral development in adolescents with childhood neglect

## *Supplemental Information*

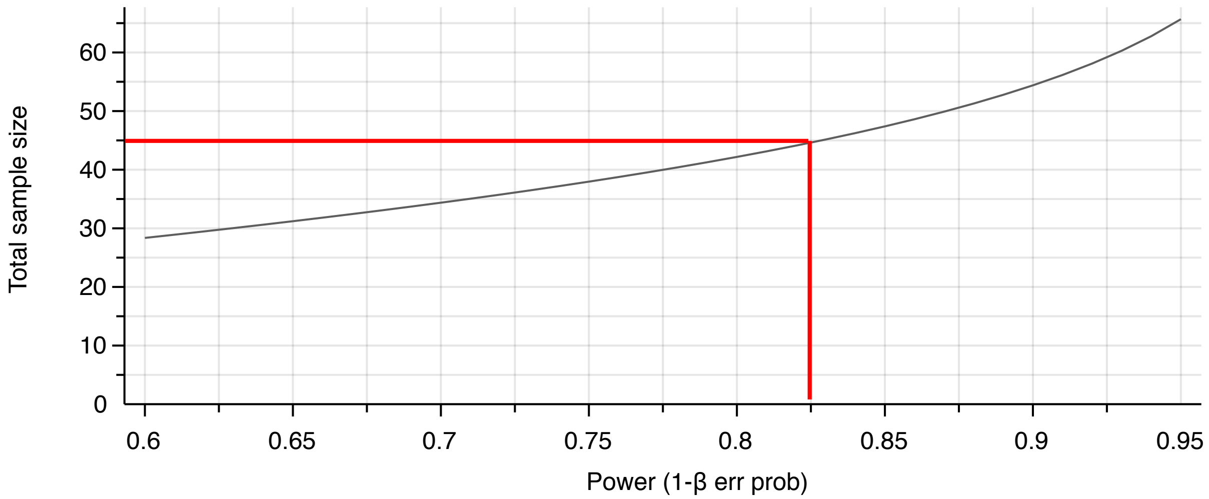


**Supplementary Figure S1.** Sample size by power plot. Calculations are for a repeated measures analysis of variance for detecting a large effect size ($\eta^2 \geq 0.14$[56]) between-group effect compared for three intervention groups (IAI, EAI, NI) with two repeated measures (baseline vs. post-intervention). The red intersecting lines show that a sample size of 45 is powered at >0.8 with alpha level of 0.05.
